# How can environmental fecal sampling support *Echinococcus multilocularis* surveillance in wild carnivores?

**DOI:** 10.1016/j.fawpar.2026.e00352

**Published:** 2026-06-27

**Authors:** Josefine Wassermann, Marin Bussi, Martina Abs, Alrik-Markis Kunisch, Martin J. Oettler, Marie Krebs, Franziska Rachel, Jonas Heck, Christine Luttermann, Heiko Schmüser, Kerstin Wernike, Carola Sauter-Louis, Hannes Bergmann, Gereon R.M. Schares

**Affiliations:** a Friedrich-Loeffler-Institut, Federal Research Institute for Animal Health, Institute of Epidemiology, Südufer 10, 17493 Greifswald - Insel Riems, Germany; b Friedrich-Loeffler-Institut, Federal Research Institute for Animal Health, Institute of Diagnostic Virology, Südufer 10, 17493 Greifswald - Insel Riems, Germany; c Friedrich-Loeffler-Institut, Federal Research Institute for Animal Health, Institute of Immunology, Südufer 10, 17493 Greifswald - Insel Riems, Germany; d Christian-Albrechts-Universität, Department of Landscape Ecology, Institute for Natural Resource Conservation, Kiel University, Olshausenstraße 75, 24118 Kiel, Germany

**Keywords:** *Echinococcus multilocularis*, Necropsy findings, Environmental contamination, Fox, Raccoon dog, Fox microsatellite typing

## Abstract

Foxes (*Vulpes vulpes*) and raccoon dogs (*Nyctereutes procyonoides*) are natural definitive hosts of *Echinococcus multilocularis*. The aim of this study was to compare *E. multilocularis* findings from the necropsy analysis of hunted animals with those from environmental fecal samples. Between November 2023 and March 2025, 84 carcasses (58 foxes, 26 raccoon dogs) were provided by volunteer hunters located in the area of two municipalities of the island Rügen in North-Eastern Germany. Analysis using the sedimentation counting technique and real-time PCR resulted in a combined positive detection of *E. multilocularis* in 37.9% (95% Confidence interval [CI] 25.5–51.6%) of foxes and in 15.4% (95% CI 4.4–34.9%) of raccoon dogs. Between February and April 2025, during 13 excursions in the same two municipalities, two-person teams collected 365 environmental fecal samples, which were targeted to be from foxes and raccoon dogs. Species identification through species-specific real-time PCR revealed that the fecal samples were from foxes (56.2%), raccoon dogs (2.7%), domestic dogs or wolves (9.0%), domestic cats or wildcats (0.6%), raccoons (0.3%), and unknown species (26.0%). *E. multilocularis* DNA was detected by real-time PCR in 18.1% (95% CI 13.1–24.1%) of fox and in 10.0% (95% CI, 0.3–44.5%) of raccoon dog feces. The examination of environmental fecal samples for *E. multilocularis* appeared to be less sensitive compared to the analysis of necropsy samples from hunted foxes and raccoon dogs. The lower proportion of *E. multilocularis* DNA in environmental fecal samples may be due to adverse environmental factors, such as degradation by UV light or leaching from precipitation. Nevertheless, environmental fecal sample collection was less labor-intensive and time-consuming and was able to confirm the presence of *E. multilocularis* in both municipalities. Restricting the analysis to environmental fecal samples containing a minimal quantity of host DNA yielded a prevalence estimate closer to that obtained from necropsy.

## Highlights

• Analyzing environmental feces showed the occurrence of *E. multilocularis.*
• The results by necropsy and environmental fecal sampling were compared.
• Fox necropsy revealed a higher apparent prevalence compared to environmental feces.
• Most positive findings occurred in unformed or partially unformed fecal samples.
• Host DNA-based environmental feces selection lead to better prevalence estimates.

## Graphical abstract

**Figure:**
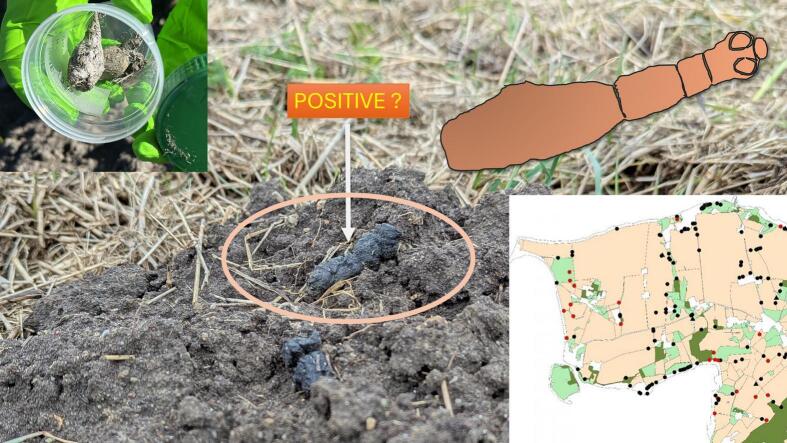

## 1 Introduction

Human alveolar echinococcosis (AE) caused by *E. multilocularis* is a zoonotic disease which is frequently reported in the southern Federal States of Germany, and less frequently in the north of Germany (Schmidberger et al., 2018). More recent data suggested that the incidence of human AE in southwest Germany is increasing and that there is an urbanization of the disease (Mueller et al., 2020) which indicates that regular surveillance programs are necessary in Germany.

Foxes are the main definitive hosts of *E. multilocularis* in temperate Europe (Kapel et al., 2006; Thompson et al., 2006). Additionally, raccoon dogs may serve as natural definitive hosts, but their importance for the lifecycle and distribution of *E. multilocularis* and AE is still under discussion (Conraths and Deplazes, 2015).

There is evidence that foxes are infected with *E. multilocularis* to a variable extent throughout Germany, with high prevalences reported in the south and moderate to low prevalences in central and northern regions of the country (Conraths and Maksimov, 2020; Oksanen et al., 2016). However, no nation-wide, standardized surveillance system is in place to enable a scientifically robust assessment of *E. multilocularis* occurrence in foxes across Germany. Available data is fragmented due to the short-term surveillance programs conducted in various federal states over several decades, often at different times (summarized in Conraths and Maksimov (2020)).

The lack of harmonized surveillance is likely due to several factors: (i) the need for hunters to voluntarily participate through fox or raccoon dog hunting, (ii) the transport of carcasses to necropsy facilities, (iii) the *E. multilocularis*-specific decontamination of carcasses by freezing several days at −70 °C, (iv) the necropsy itself, (v) the collection of intestinal mucosa, and (vi) the parasitological analysis, which requires time-consuming and costly methods. In addition, outbreaks of other diseases of veterinary importance, like avian influenza or African swine fever, are binding resources needed for *E. multilocularis* surveillance programs.

More recent studies in other countries like France (Da Silva et al., 2020; Da Silva et al., 2024; Umhang et al., 2016), Turkey (Gürler et al., 2018), Italy (Massolo et al., 2018) and Canada (Liccioli et al., 2015) showed that environmental fecal sampling is an option for a non-invasive and less costly sampling of definitive hosts. Therefore, this study aimed to compare *E. multilocularis* findings in environmental fecal samples from foxes and raccoon dogs with those obtained from the necropsy of hunted animals. Additionally, we examined the relationship between the appearance of fox samples at the time of collection, and the detection of fox DNA and *E. multilocularis* DNA. Furthermore, fox-derived environmental samples were analyzed using fox microsatellite typing to investigate whether individual foxes contributed multiple times to the environmental sample set.

## 2 Material & methods/methods

### 2.1 Study area

**Fig. 1:**
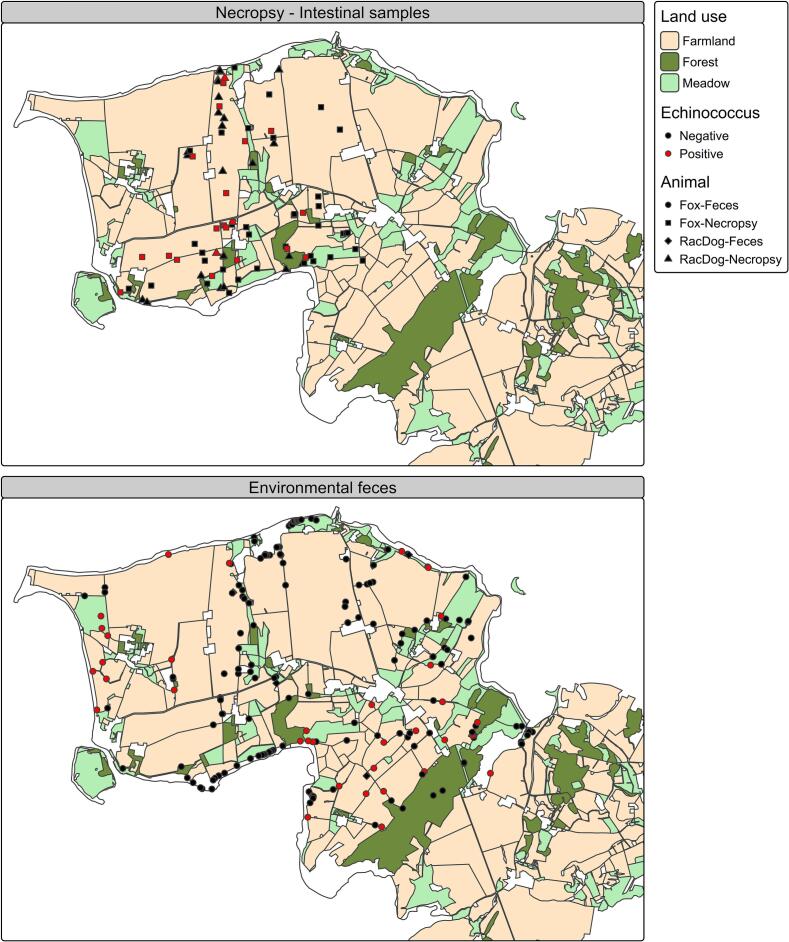
*E. multilocularis* detection in foxes and raccoon dogs in two municipalities located on the island Rügen, North-Eastern Germany. Necropsy – Intestinal samples: Results on *E. multilocularis* obtained by direct detection (Sedimentation-Counting-Technique) and by DNA detection after intestinal swabbing and examination of feces collected from the terminal rectum of hunted foxes (Fox-Necropsy) and raccoon dogs (RacDog-Necropsy). Environmental feces: Detection of *E. multilocularis* DNA in fecal samples collected from foxes (Fox-Feces) and raccoon dogs (RacDog-Feces) in the environment. Land-use categories (Land use) are colored: light brown = Farmland: arable land; light green = Meadow: grassland; pasture; dark green = Forest.

The study area was located in two municipalities (Schaprode and Trent, covering 19.8 km^2^ and 35.4 km^2^, respectively) on a peninsula at the western shore site of the island Rügen in the German Federal State of Mecklenburg-Western Pomerania (between latitudes 54.493577 and 54.554262, and between longitudes 13.139436 and 13.302601). The study area was confined by Baltic sea-water bodies in the north, the west and the south. The study area consisted mainly of farmland (i.e. arable land) which was interspersed by grassland and pastures. In the eastern part a larger proportion of the area was covered by forest (Fig. 1).

### 2.2 Sampling

#### 2.2.1 Hunting, storage, transport and necropsy of carcasses

Wild carnivores that were hunted or died for other reasons were collected and stored at −20 °C or −80 °C at specific collection sites on the island Rügen. In intervals of 3–6 weeks, carcasses were gathered from collection sites and transported at ambient temperatures to the Friedrich-Loeffler-Institut for monitoring of wildlife diseases. Foxes and raccoon dogs were necropsied after at least 1 week of storage at −80 °C to minimize the risk of *E. multilocularis* infection for persons involved in the necropsy.

After storage, the carcasses were thawed, and various sample types were collected during necropsy. The entire small intestines of the animals were incised longitudinally using scissors and the intestinal contents superficially removed. Sampling followed protocols previously described (Kunisch et al., 2025). After the initial swabbing, intestinal mucosa was scraped and collected; in addition, a fecal sample from the terminal rectum was collected (Kunisch et al., 2025).

#### 2.2.2 Collection of environmental fecal samples

A total of nine persons were involved in environmental collection of feces from the study area. Only two of these persons were hunters; these two participated only in a single tour. The remaining persons participated in one to seven tours. In total, the two-person teams did *n* = 13 different tours in the area of the two Rügen municipalities. Mean distances covered by the circular tours were 8.6 km (+/− 1.5 km standard deviation). Each tour lasted between 5 and 6 h, except the very first which lasted about 2 h, due to freezing temperatures that day. None of the tracks was repeated. Teams usually followed established paths, walked along ditches or on small dikes. Occasionally, meadows, grassland, pastures or fields were crossed. Areas around field trees, or small elevations received special attention, thus implementing search recommendations from local hunters. Private, fenced areas like gardens or farm premises were excluded from sampling. Given the systematic nature of the sampling approach and the coverage of diverse habitats within the study area, this collection approach was assumed to provide a representative sample of relevant areas across the entire targeted region.

The identification of fox and raccoon dog feces was based on the recognition of their species-specific visual appearance, which had been rehearsed prior to field collections through inspection of image examples. Participants were required to wear gloves during collection to avoid touching feces with their bare hands. Since we wanted to determine whether the appearance of fecal samples at the time of collection (color, freshness, consistency, form, visible contents) provided clues about the amount of host DNA or the possibility of detecting *E. multilocularis* DNA, the teams were asked to take photos of each sample prior to collection using smartphones. The precise geographic location was recorded using Garmin GPSMAP 65s Outdoor Navigation equipment (Garmin, Schaffhausen, Switzerland). For occupational safety reasons, the feces were deep-frozen at −80 °C for at least one week after collection and before analyses began (Eckert et al., 2001).

### 2.3 Sedimentation counting technique

**Fig. 2:**
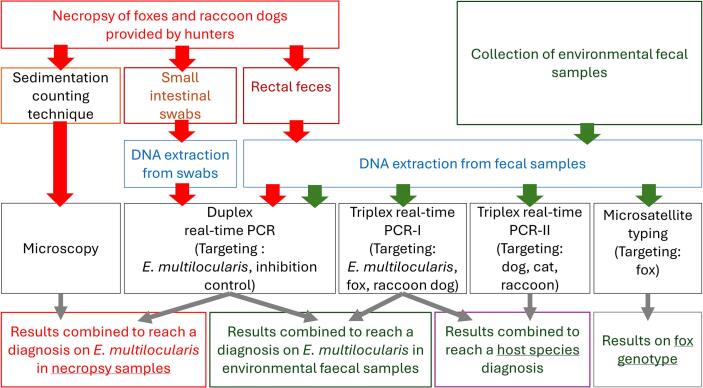
A flowchart illustrating the various steps and methods applied to examine samples collected during necropsy, as well as fecal samples from the environment.

Sedimentation Counting Technique (SCT, Fig. 2) was methodologically based on an earlier description (Hofer et al., 2000), but slightly modified (Kunisch et al., 2025). In brief, the intestinal mucosa, scraped off and filled into a beaker, was stored frozen (−20 °C) until use. For SCT, the entire mucosal sample was transferred through a strainer into funnels and resuspended in physiological saline solution. After three sedimentation-resuspension cycles, the supernatant was decanted to 40–80 ml, and the sediment fraction was examined in aliquots in square Petri dishes under a transmission light stereomicroscope at a magnification of 120× and the detailed number of observed worms was recorded. SCT results, displayed as infection intensity, were categorized according to their worm count as follows: > 1000 (++++), 101–1000 (+++), 11–100 (++), 1–10 (+).

### 2.4 DNA extraction of intestinal swabs, fecal samples taken during necropsy and environmental fecal samples

DNA was extracted from small intestinal swabs (Fig. 2) following the protocol previously described (Kunisch et al., 2025). A 2 ml reaction tube containing the tip of a swab was filled with 400 μl of PBS and 25 μl of a proteinase K solution (0.01 mol/l in Tris-HCl pH 7.5; Sigma–Aldrich, Deisenhofen, Germany), vortexed and incubated in a thermomixer (56 °C, 800 rpm, 10 min). After incubation, the tube was centrifuged (2000 ×*g*, 30 s). 50 μl of the supernatant was added to 200 μl of T1 buffer supplemented with 25 μl of proteinase K solution provided with the NucleoMagTissue Kit (Macherey-Nagel, Düren, Germany) and incubated in a thermomixer (56 °C, 550 rpm, overnight). For DNA extraction, 200 μl of the supernatant was used. DNA extraction was performed by using the NucleoMag Tissue Kit (Macherey-Nagel, Düren, Germany).

DNA was extracted from both types of fecal samples (Fig. 2), those collected during necropsy and those gathered during environmental collection tours, using the method previously described (Kunisch et al., 2025). Fecal samples were usually used as collected. However, if samples were solid or dry, a similar volume of sterile PBS was added to the sample and used to prepare a sample suspension before continuing the extraction process. Each sample suspension (3 g) was mixed with 0.8 g of 0.5 mm, 0.6 g of 2 mm and 1 g of 0.1 mm zirconium oxide beads (BioSpec Products Inc., Bartlesville OK, USA), as well as 6 ml of homogenization and lysis buffer (100 mM Tris HCl pH 8.0, 5 mM EDTA pH 8.0, 0.2% SDS, 200 mM NaCl) and 2 ml of 5 M NaCl in a 15 ml Sarstedt screw cap tube, secured by Parafilm (Sigma–Aldrich, Deisenhofen, Germany). The sample was then bead-beaten using a FastPrep homogenizer (MP Bio, Santa Ana CA, USA). Shredding was carried out three times for 60 s each time at a maximum speed of 6.0 m/s. The tubes were then centrifuged for 10 min at 3000 ×*g* without brake, and the supernatants of the homogenates were recovered and processed further. To inactivate potentially inhibitory components, 200 μl of the homogenate was mixed with 200 μl of Stool Transport and Recovery Buffer (S.T.A.R. buffer; Roche Diagnostics, Mannheim, Germany), incubated for 15 min at 95 °C in a thermomixer (500 rpm) and subsequently centrifuged (4 °C, 14.000 ×g, 5 min). For DNA extraction, 200 μl of the supernatant was used. In fecal samples, DNA extraction was performed via the NucleoMagVet Kit (Macherey-Nagel, Düren, Germany), using a King Fisher 96 Flex (Thermo Fisher Scientific, Braunschweig, Germany) according to kit manuals.

Finally, 100 μl purified DNA extraction were eluted. Every 8th sample in the extraction plate represented a negative processing control. Each run included a negative extraction control used throughout DNA extraction.

### 2.5 Real-time PCRs for *E. multilocularis* and host DNA

#### 2.5.1 Duplex real-time PCR

All DNA samples were tested for the presence of *E. multilocularis* DNA via MGBqPCR, which targets the small ribosomal RNA gene (s-rRNA) (Isaksson et al., 2014; Kunisch et al., 2025) along with an internal control plasmid DNA (duplex real-time PCR; Fig. 2). All the DNA samples (including negative processing controls) were tested by adding 7.5 μl of the QuantiTect Multiplex PCR NoROX Kit (200 × 50 μl reactions, QIAGEN, Hilden, Germany), 400 nM each of the primers EmMGB_F and EmMGB_R, and 133 nM of the hydrolysis probe EmMGB_P-VIC (Maksimov et al., 2019). The reactions were run in a total volume of 15 μl, including 2 μl of template DNA. The duplex real-time PCR was carried out in a Bio-Rad CFX96 Real-Time Detection System (Hercules, Bio-Rad Laboratories GmbH, Munich, Germany) at 50 °C for 2 min, followed by an initial denaturation step at 95 °C for 10 min and 45 amplification cycles at 95 °C for 15 s, followed by annealing and elongation at 60 °C for 1 min. Fluorescence was measured at the end of each cycle. For all DNA samples assessed for *E. multilocularis* by the duplex real-time PCR (Fig. 2), cycle of transition values (Ct) of <39 were regarded as positive, Ct 39–41 inconclusive and Ct >41 negative. These cut-offs had been applied previously (Kunisch et al., 2025) and were based on those proposed by others (Isaksson et al., 2014; Maksimov et al., 2019).

To assess the presence of inhibitory components in the duplex real-time PCR, heterologous plasmid DNA resembling the gene that encodes enhanced green fluorescent protein (EGFP) (Hoffmann et al., 2006) was added to the reaction mixture, which included the primers EGFP1-F and EGFP2-R and the probe EGFP1 (Schares et al., 2020). In samples where neither *E. multilocularis* nor internal control DNA was amplified (cut-off Ct <38), inhibition was assumed, and a negative result was regarded as not valid.

#### 2.5.2 Triplex real-time PCR-I and triplex real-time PCR-II

Environmental fecal samples were further assessed by the triplex real-time PCR-I (Fig. 2), applying primers and probes to simultaneously detect *E. multilocularis*-DNA as well as fox and raccoon dog DNA. For the detection of *E. multilocularis* DNA in the triplex real-time PCR-I, the same cut-offs as described for the duplex real-time PCR were applied. Consequently, a sample with a Ct value <39 was regarded as positive, Ct value of 39–41 as inconclusive and a Ct value >41 as negative.

To detect fox DNA, primers and a FAM-labelled probe targeting the cytochrome *b* (*cyt*b) gene published previously were used (Knapp et al., 2016). Because no primers and probes to specifically detect raccoon dog DNA were found in literature, novel primers and a probe were established based on the sequence of the raccoon dog *cyt*b gene (*Nyctereutes procyonoides* complete mitogenome, 16713 bp, GenBank: NC_013700) using Np_mt_F, 5′- TCAATCCTAATCCTAGTATTCACC-3′ and Np_mt_R, 5’-AACATGCATTGACTAAGTGGT-3′ as primers and Np_mt_P, TexasRed-5’-CCACACATCCAAACAACGTG-3’-BBQ-650 as a probe (metabion international AG, Planegg, Germany).

The reactions of the triplex real-time PCR-I were run in a total volume of 15 μl using the QuantiTect Multiplex PCR NoROX Kit, including 2 μl of template DNA. All primers in the triplex real-time PCR-I were used at a final concentration of 400 nM and all probes at a final concentration of 220 nM.

To investigate the host species identity further, a triplex real-time PCR-II was applied using primers and probes for testing for dogs, cats or raccoons (Fig. 2). For this PCR, the primers and probes for dogs and cats targeting the *cyt*b gene were applied as previously reported (Knapp et al., 2016). To detect raccoon DNA, we applied previously published primers and a probe targeting the cytochrome *c* oxidase subunit I (COI) gene of raccoons (Koyama et al., 2026).

The reactions of the triplex real-time PCR-II were run in a total volume of 15 μl using the QuantiTect Multiplex PCR NoROX Kit, including 2 μl of template DNA. All primers in the triplex real-time PCR-II were used at a final concentration of 400 nM and all probes at a final concentration of 220 nM.

The triplex real-time PCR-I and the triplex real-time PCR-II had been validated in unpublished experiments using DNA isolated from feces of red foxes (*Vulpes vulpes*, *n* = 4), raccoon dogs (*Nyctereutes procyonoides*, *n* = 4), raccoons (*Procyon lotor*, *n* = 4), domestic dogs (*Canis lupus familiaris*, *n* = 3), wolves (*Canis lupus*, *n* = 4), wildcats (*Felis silvestris*, *n* = 4), domestic cats (*Felis catus*, *n* = 4), badgers (*Meles meles*, *n* = 3), and pine marten (*Martes martes*, *n* = 2) (Additional file 1). These validation experiments revealed that DNA of wolves and wildcats reacted in the real-time PCR previously established for the detection of dogs and cats, respectively (Knapp et al., 2016). Because also the other species-specific PCRs showed occasionally some cross-reactivity (Additional file 1), a cut-off of Ct <38 was used to confirm the animal species from which a fecal sample originated. If cross-reactivity was observed for samples with Cts below the selected cut-off, species diagnosis was reached by assessing the difference in Ct values between both species-specific real-time PCRs (ΔCt). If the Ct value in one PCR was at least 3 Cts lower compared to the remaining one (ΔCt >3), this result was used for species confirmation. In cases in which for the PCR results a ΔCt <3 was recorded, no species diagnosis could be reached.

### 2.6 Microsatellite typing of fox DNA

For the microsatellite typing of fox DNA (Fig. 2), eight microsatellite markers (AHT121, AHT137, INU055, AHTh171, C04.140, FH2848, REN169O18, CPH11) and one sex marker (K9-AMELO) were used; primer sequences had previously been published (Da Silva et al., 2024). The forward-primer of each of the primer pairs was labelled by a fluorophore: FAM in case of AHT121, AHT137, and INU055; HEX in case of AHTh171, REN169O18, and FH2848; ATTO550 in case of CPH11, C04.140, and the sex marker K9-AMELO. A mixture of all primers (0.2 pmol/μl final concentration per primer) was applied in a multiplex PCR using the 2× Multiplex PCR Master Mix (QIAGEN, Hilden, Germany). The PCR using 5 μl template of fox-DNA in 25 μl total volume was carried out in a Mastercycler Nexus (Eppendorf, Hamburg, Germany) after an initial denaturation step at 95 °C for 15 min, using 35 cycles (94 °C, 30 s; 61 °C, 3 min; 72 °C 30 s) followed by an elongation step of 60 °C, 30 min. After the PCR, 1 μl of each sample was mixed with 10 μl HiDi® formamide (Applied Biosystems) and 0.3 μl of ROX500 (ROX 500, Applied Biosystems) and denatured over 5 min at 95 °C. After denaturation, fragment sizes were assessed on a capillary sequencer (Hitachi 3500 Genetic Analyzer, ABI), and location of peaks analyzed using the Geneious Prime software (version 2025.1.3).

### 2.7 Statistical analysis

Non-equivocal *E. multilocularis* results in the two real-time PCRs (duplex real-time PCR, triplex real-time PCR-I) were combined using the following decision tree. In case, a positive *E. multilocularis* PCR result was obtained in only one of the two PCRs, the sample was regarded as positive. In case, the result was inconclusive in one of the PCRs and negative in the remaining PCR, the sample was regarded as negative.

The results were analyzed using R version 4.5.1 (R Foundation for Statistical Computing, Vienna, Austria; http://www.R-project.org), and RStudio 2025.05.1. The package “binom” was used to determine 95% CI. The Spearman's rank correlation coefficient was calculated using the package “smplot2”. Figures were assembled via R, version 4.5.1 (packages “ggplot2”, “smplot2” and “cowplot”). Fisher's exact statistical tests were performed using the “fisher.test” function from the R package “stats”. For mapping the packages “tmap” and “tmaptools” were used. Land-use data was used from OpenStreetMap at https://download.geofabrik.de/europe/germany/mecklenburg-vorpommern.html; last accessed on 21 April 2025.

Kappa value (*κ*) was calculated via an online tool (http://vassarstats.net, last accessed 2 June 2026). Kappa values were interpreted as follows: *κ* < 0 = poor agreement, 0 < *κ* < 0.20 slight, 0.21< *κ* < 0.40 = fair, 0.41 < *κ* < 0.60 = moderate, 0.61 < *κ* < 0.80 = substantial, 0.81 < *κ* < 1.00 = almost perfect (Landis and Koch, 1977).

## 3 Results

### 3.1 Results for hunted foxes and raccoon dogs

Between November 2023 and March 2025, a total of 58 foxes (35 male and 23 female) and 26 raccoon dogs (7 male and 19 female) were provided by five hunters from the study area, comprised of the two municipalities, Schaprode and Trent. Most of the animals (54/58 [93%] foxes and 24/26 [92%] raccoon dogs) were supplied by a single hunter active in the study area.

Of the 58 foxes examined, 18 (31.0%; 95% CI 19.5–44.5%) were positive in SCT (*n* = 8, +; *n* = 6, ++; *n* = 2, +++; n = 2, ++++). In the duplex real-time PCR on swabs, 22/58 (37.9%; 95% CI 25.5–51.6%) and on feces from the terminal rectum 21/56 (37.5%; 95% CI 24.9–51.5%) of the foxes tested positive (for two foxes no rectal feces were available; one was negative and the other positive in both, SCT and swab real-time PCR). All SCT positive foxes were positive in at least one of the two real-time PCRs, on DNA from swabs and from rectal feces. The remaining four positive foxes were SCT negative but tested positive in both real-time PCRs, on DNA from swabs and from rectal feces. Three SCT negative foxes, in which only either the fecal or the swab PCR revealed a positive result were regarded as negative. Overall, the examination for *E. multilocularis* after necropsy (SCT, real-time PCR on intestinal swabs and fecal samples) revealed a total number of 22/58 (37.9%; 95% CI 25.5–51.6%) positive foxes (Fig. 1, Additional file 2: Table S1).

Of the 26 raccoon dogs examined 3 (11.5%; 2.5–30.2%) were positive by SCT (*n* = 1, +; n = 1; ++; n = 1, +++). An additional racoon dog was regarded as positive, because both, the intestinal swab as well as the fecal DNA tested positive, although SCT was negative. Based on the duplex real-time PCR results on swabs 4/26 (15.4%; 95% CI 4.4–34.9%) and on feces from the terminal rectum 4/25 (16.0%; 95% CI 4.5–36.1%) of the raccoon dogs tested positive (for one raccoon dog no rectal feces were available; this animal was positive in both, SCT and swab real-time PCR). Overall, in raccoon dogs, a total of 4/26 (15.4%; 95% CI 4.4–34.9%) tested positive (Fig. 1, Additional file 2: Table S1).

When the *E. multilocularis* results of SCT, duplex real-time PCR and triplex real-time PCR-I were compared such values were reached, suggesting substantial agreement in all comparisons (SCT vs duplex real-time PCR on swabs: *κ* = 0.79, 95% CI 0.65–0.94; SCT vs duplex real-time PCR on feces: *κ* = 0.66, 95% CI 0.48–0.85; duplex real-time PCR on swabs vs duplex real-time PCR on feces: *κ =* 0.77, 95% CI 0.62–0.92; Additional file 2: Table S2).

### 3.2 Results for environmental fecal samples

#### 3.2.1 Sampling and species diagnostics

A total of 365 fecal samples were collected. Species diagnostics using real-time PCR revealed that 205 (56.2%) of these samples were from foxes and 10 (2.7%) from raccoon dogs, 1 from a raccoon (0.3%), 33 from dogs or wolves (9.0%) and two from cats or wildcats (0.6%). For the remaining 114 (31.2%) samples no species diagnosis could be reached (due to no signals or ΔCt results <3 in two species-specific real-time PCRs which did not allow to decide on the species; Additional file 2: Table S3).

#### 3.2.2 PCR analysis of *E. multilocularis* in environmental fecal samples

*E. multilocularis* DNA was detected by real-time PCR in 37/204 (18.1%; 95% CI 13.1–24.1%) of the fox feces and in 1/10 (10.0%; 95% CI 0.3–44.5%) of the raccoon dog feces (combined results by duplex real-time PCR and triplex real-time PCR-I; Table 1; Additional file 2: Table S3). All samples from dogs, cats and the raccoon tested negative for *E. multilocularis* DNA. One of the fox samples was inhibited according to the internal control PCR and was excluded from the data analysis. In one *E. multilocularis* positive sample the animal species from which the sample originated could not be determined (no Ct). In the case of foxes, the prevalence estimated by environmental feces (18.1%) was statistically lower as compared to the prevalence estimate obtained by necropsy (37.9%; Fisher Exact Test, *p* = 0.002). In all other host species, the numbers of animals were too low for statistical analysis.

**Table 1:** Results of two multiplex real-time PCRs on *E. multilocularis* DNA in environmental samples. In the duplex real-time PCR, *E. multilocularis* was assessed including an inhibition control. In the triplex real-time PCR-I, the presence of host (fox or raccoon dog) and DNA *E. multilocularis* DNA was analyzed in parallel.

Animal species	Test		Triplex real-time PCR-I			
		Test result	Positive	Inconclusive	Negative	Total
Fox	Duplex real-time PCR	Positive	23	5	9	37⁎
		Inconclusive	0	0	2	2
		Negative	0	1	165	166
		Fox, Total	23	6	176	205
Raccoon-dog	Duplex real-time PCR	Positive	0	0	1	1⁎
		Inconclusive	0	0	0	0
		Negative	0	0	9	9
		Raccoon-dog Total	0	0	10	10
Dog/wolf	Duplex real-time PCR	Positive	0	0	0	0
		Inconclusive	0	0	0	0
		Negative	0	0	33	33
		Dog Total	0	0	33	33
Cat/wildcat		Positive	0	0	0	0
		Inconclusive	0	0	0	0
		Negative	0	0	2	2
		Cat Total	0	0	2	2
Raccoon	Duplex real-time PCR	Positive	0	0	0	0
		Inconclusive	0	0	0	0
		Negative	0	0	1	1
		Raccoon Total	0	0	1	1
Unknown species	Duplex real-time PCR	Positive	0	0	1	1⁎
		Inconclusive	0	0	0	0
		Negative	0	0	113	113
		Unknown Total	0	0	114	114

⁎ These samples were regarded as positive. Samples which had tested inconclusive in one of the PCRs but negative in the remaining PCR were regarded as negative.

The duplex real-time PCR (*E. multilocularis* and inhibition control) detected more *E. multilocularis* positive samples, and 5 samples inconclusive in the triplex real-time PCR-I (*E. multilocularis*, fox) were confirmed for the presence of *E. multilocularis* DNA (Table 1). Out of the samples negative for *E. multilocularis* in the triplex-PCR-I, 9 samples from foxes, one sample from a raccoon dog and one sample from an unknown animal species tested positive for *E. multilocularis* DNA in the duplex real-time PCR (Table 1). Three further samples (from foxes only), inconclusive in one of the PCRs tested negative in the other PCR (Table 1). Overall, the agreement between both PCR analyses on *E. multilocularis* DNA in the duplex real-time PCR and the triplex real-time PCR-I for samples for which the host species could be determined was substantial (Unweighted *κ* = 0.704; 95% CI 0.573–0.836). If the analysis was restricted to foxes a similar level of agreement was reached (Unweighted *κ* = 0.708; 95% CI 0.576–0.841).

**Fig. 3:**
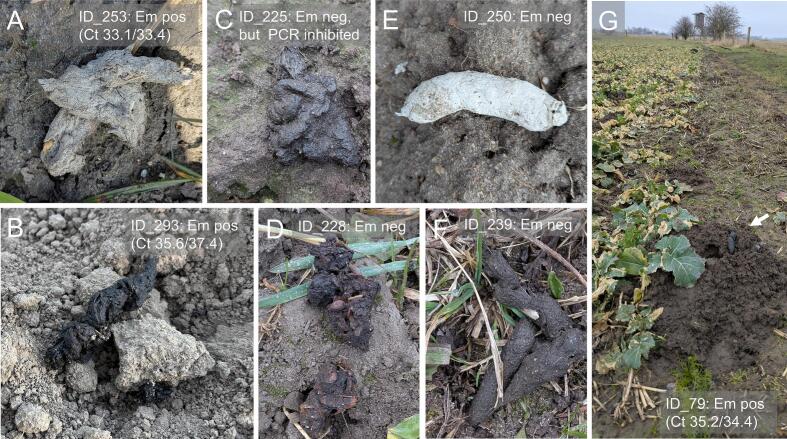
Example images for environmental fox fecal samples later tested *E. multilocularis* real-time PCR positive (Em pos) or negative (Em neg) (sample identification numbers [IDs] as well as in positive cases the Ct values for *E. multilocularis* DNA in the duplex real-time PCR and in the triplex real-time PCR-I are provided on each of the images). In case of ID_225 the *E. multilocularis* duplex real-time PCR was not valid, because the internal control had a Ct > 38.0 (i.e. the PCR was regarded as inhibited). **A, B, C, D** – Samples scored as amorphous or partially amorphous, **E, F, G** – Samples scored as well formed, **A, E** – Samples of white color or mixed color, dominated by white or grey, **B, C, D, F, G** – Samples scored as samples of dark color, **G** – Typical location of a fox fecal sample adjacent to a field on a vole mound or molehill (arrow).

Furthermore, we wanted to determine whether the appearance of fecal samples at the time of collection (color, freshness, consistency, form, visible contents) provides clues about the amount of host DNA or the possibility of detecting *E. multilocularis* DNA. The analysis was restricted to the evaluation of photos taken from *n* = 182 fox samples as determined by a fox-specific real-time PCR (Fig. 3). Samples with a white color or mixed colors dominated by white or grey had significantly less often Ct values <27.0 in the fox-DNA specific real-time PCR as compared to samples colored dark (Table 2; Fisher exact test *p* = 0.01). In case of detection of *E. multilocularis*-DNA by real-time PCR, it was observed that samples with an unformed appearance (complete or partial) were significantly more often *E. multilocularis* positive as compared to formed samples (Table 3; Fisher exact test *p* = 0.025).

**Table 2:** Fox-DNA real-time PCR results stratified by a cut-off of Ct 27 relative to the appearance of fecal samples at the time of collection (color, freshness, consistency, form, visible contents). Samples colored dark or in which black, blue or green colors dominated, tested more often Ct <27 in the fox-DNA real-time PCR (Fisher exact test, *p* = 0.01). Analysis was restricted to n = 182 fox-DNA positive samples for which a photo had been taken at the point of sampling.

Criterion	Appearance of fox fecal samples	Real-time PCR for fox DNA		*P*-value
		Ct <27	Ct ≥27	
Color of feces	Dark colors (brown, black, mixed colors dominated by black, blue or green)	48 (47.5%)	53 (52.5%)	0.01
	White color or mixed colors dominated by white or grey	23 (28.4%)	58 (71.6%)	
Freshness	Fresh	17 (42.5%)	23 (57.5%)	ns
	Not fresh	54 (38.0%)	88 (62.0%)	
Consistency	Disintegrated	13 (50.0%)	13 (50.0%)	ns
	Compact	58 (37.2%)	98 (62.8%)	
Form	Well formed	62 (37.8%)	107 (62.2%)	ns
	Unformed (complete or partially)⁎	9 (50.0%)	9 (50.0%)	
Visible contents	Hair	34 (34.7%)	64 (65.3%)	ns
	Feathers	4 (22.2%)	14 (77.8%)	
	Hair and feathers	3 (100%)	0 (0%)	
	Unknown/other	30 (47.6%)	33 (52.4%)	
Total	Total	71 (39.0%)	111 (61.0%)	

⁎ The majority of samples characterized as unformed had no disintegrated character (15/18; 83.3%).

**Table 3:** Proportions of *E. multilocularis* real-time PCR positive fox fecal samples in relation to the appearance of fecal samples at the time of collection (color, freshness, consistency, form, visible contents). Unformed or partially unformed samples were significantly more often *E. multilocularis*-DNA-positive as compared to formed samples (Fisher exact test, *p* = 0.025). Analysis was restricted to *n* = 181 fox DNA positive samples for which a photo had been taken at the point of sampling. One of the fox samples was inhibited in the *E. multilocularis* real-time PCR according to the internal control PCR and was excluded from this data analysis.

Criterion	Appearance of fox fecal samples	Real-time PCR for *E. multilocularis* DNA		*P*-value
		Positive	Negative	
Color of feces	Dark colors (brown, black, mixed colors dominated by black, blue or green)	22 (22.0%)	78 (78.0%)	ns
	White color or mixed colors dominated by white or grey	13 (16.0%)	68 (84.0%)	
Freshness	Fresh	6 (15.4%)	33 (84.6%)	ns
	Not fresh	29 (20.4%)	113 (79.6%)	
Consistency	Disintegrated	7 (26.9%)	19 (73.1%)	ns
	Compact	28 (18.1%)	127 (81.9%)	
Form	Well-formed	28 (17.1%)	136 (82.9%)	0.025
	Unformed (completely or partially)	7 (41.2%)	10 (58.8%)	
Visible contents	Hair	16 (16.3%)	82 (83.7%)	ns
	Feathers	3 (16.7%)	15 (83.3%)	
	Hair and feathers	1 (33.3%)	2 (66.7%)	
	Unknown/other	15 (24.2%)	47 (75.8%)	
Total	Total	35 (19.3%)	152 (80.7%)	

Note: The majority of samples characterized as unformed had no disintegrated character (14/17; 82.4%).

#### 3.2.3 Microsatellite typing to detect repeated sampling of individual foxes

**Fig. 4:**
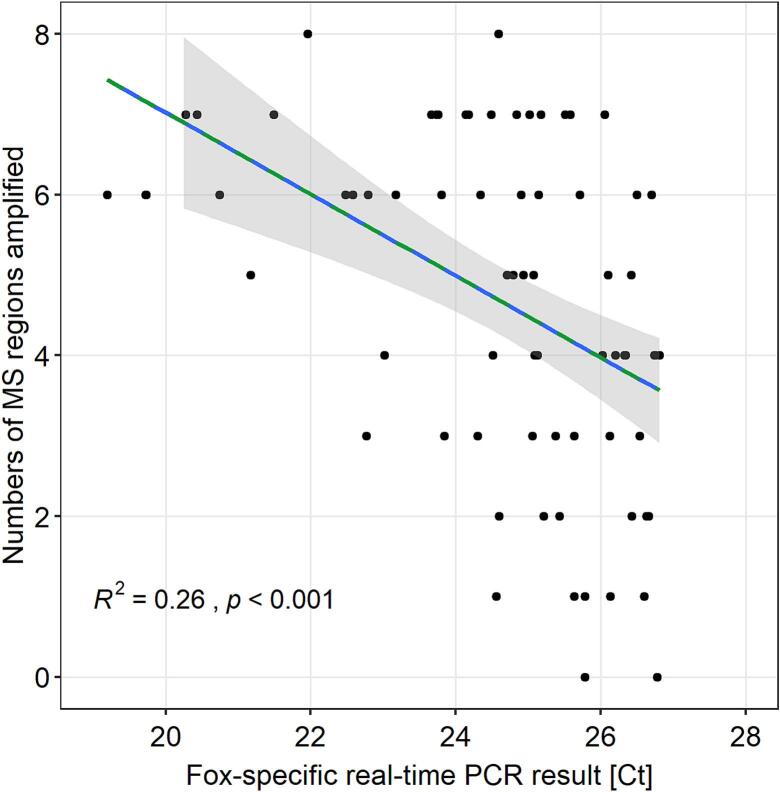
Correlation of Ct value in the fox-specific real-time PCR and number of amplified genotyping marker regions. The correlation is weak as illustrated by the coefficient of determination (R^2^) of 0.26 for 76 positive samples in the fox-DNA real-time PCR (Spearman's rank correlation coefficient). All samples in which 5 or more loci had been typed were regarded as successfully genotyped in this study.

Titration experiments with fox DNA showed that in samples with Ct values ≥27 in the fox-specific real time PCR, the chances of amplifying a sufficient number of microsatellite-typing markers were very low. Out of the 205 fox samples (including one PCR inhibited sample), 76 revealed in the fox-specific real time PCR Ct values of <27 and these samples we subjected to microsatellite genotyping. The median number for amplified regions of the 9 markers (8 markers for individual typing and 1 marker to determine sex) was 6 (Minimum *n* = 0, Maximum *n* = 9). Numbers of markers amplified correlated with the Ct values observed in the fox-specific real-time PCR (R^2^ = 0.26; Fig. 4). The marker to determine sex of foxes (K9-AMELO-1) revealed 28 samples from males and 26 samples from females, while in 29 samples the sex marker was not amplified.

There was not a single pair of samples with 100% identical typing patterns. However, since some peaks observed by capillary electrophoresis during microsatellite typing can be misinterpreted, a very similar but not identical profile could suggest that the samples originated from the same animal. To find circumstantial evidence that some of the samples came from a single animal, samples for which data on at least ≥5 typing markers were available were compared with other samples. If at least 75% of the genotyping alleles were identical and the sex determination yielded an identical result, the samples were considered suspicious for originating from the same animal.

For 21 male foxes with data for ≥5 typing markers, in total 8 pairs were identified which could represent identical animals (in the following referred to as suspicious pairs) (Table 4). In case of one pair, both samples tested *E. multilocularis* positive. In case of another pair, only one of the samples tested *E. multilocularis* positive by real-time PCR. In the remaining pairs, each sample had tested negative for *E. multilocularis*. Out of 14 female foxes with data for ≥5 typing markers, a single suspicious pair of samples (Table 4; both *E. multilocularis* negative) was identified. Some of the individual samples were observed in multiple pairs with very related microsatellite patterns (only in male foxes; at least 75% of the genotyping alleles were identical). Such pairs were combined into clusters. Cluster 1 consisted of three samples observed two times in various pairs (Table 4). Cluster 2 consisted of four samples of which two were observed in more than one pair of this cluster. The remaining clusters, consisted of a single pair only (Table 4). For each pair of samples, the geographic distances between sampling locations were determined (Table 4). The distances determined ranged from 0.08 km to 3.34 km. The majority (6/9) of the suspicious pairs with very related microsatellite patterns consisted of samples collected from locations less than 1 km apart (Table 4).

**Table 4:** Successfully typed samples occurring in multiple pairs are indicated in bold. Pairs in which part of samples occurred repeatedly were joined into clusters. It was suspected that all samples from a single cluster could have derived from a single individual.

Cluster	Sex	Pair	Allele identity	Distance between feces collection sites (km)	*E. multilocularis* positive (Ct value in in duplex real-time PCR and triplex real-time PCR-I, respectively)
1	Male	**ID_155** / **ID_158**	83%	0.08	None
1	Male	**ID_155** / **ID_173**	92%	0.76	None
1	Male	**ID_158** / **ID_173**	75%	0.8	None
2	Male	**ID_240** / **ID_372**	80%	3.11	None
2	Male	ID_270 / **ID_372**	88%	3.34	None
2	Male	ID_234 / **ID_240**	82%	0.79	None
3	Male	ID_127 / ID_302	83%	0.85	ID_302 (31.16; 31.37)
4	Male	ID_43 / ID_44	77%	0.09	ID_43 (32.76; 35.90) ID_44 (33.27; 35.72)
5	Female	ID_180 / ID_162	75%	1.07	None

#### 3.2.4 Proportion of *E. multilocularis* positives in fox-samples successfully characterized by microsatellite typing

A total of 41 fox samples were successfully characterized by microsatellite typing, i.e. ≥ 5 typing markers were determined in these samples (*n* = 21 in males, *n* = 14 in females, *n* = 6 in those, where the sex-marker had not been amplified). After identifying pairs of samples that might have originated from a single individual, only one sample from each pair was retained. If a pair consisted of one positive and one negative sample, the positive sample was retained. After excluding putative duplicates, the final list comprised 33 samples, of which 9/33 (27.3%; 95% CI 13.3–45.5%) had tested positive; 5/14 (35.7%; 95% CI 12.8–64.9%) in males, 2/13 (15.4%; 95% CI 1.9–45.5%) in females, and 2/6 (33.3%; 95% CI 4.3–77.7%) for individual foxes whose sex could not be determined.

#### 3.2.5 Proportion of *E. multilocularis* positives in relation to the Ct value in the fox-specific real-time PCR

**Fig. 5:**
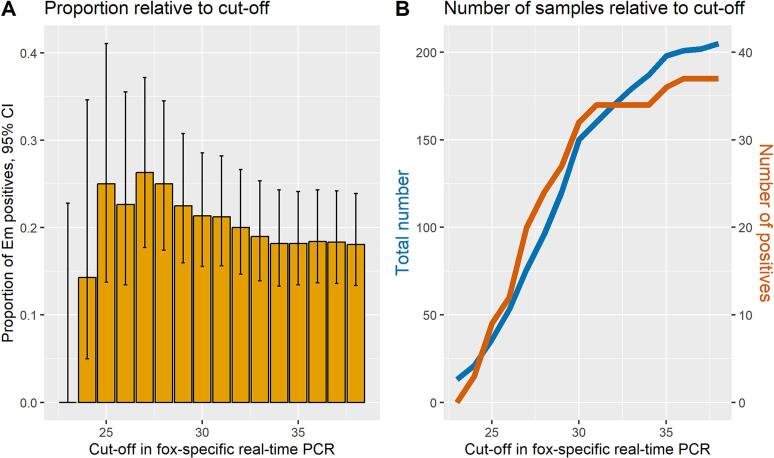
Impact of using a minimum acceptable Ct value in fox-specific real-time PCR as a cut-off for deciding on the inclusion of fox samples for prevalence estimation. (A) Proportion of *E. multilocularis* positive fox fecal samples from the environment relative to the minimum acceptable Ct in the fox-specific real-time PCR as an inclusion criterion, (B) Number of samples analyzed and number of fox samples positive in the *E. multilocularis* (Em) real-time PCR relative to the cut-off applied. **Table:**

The Ct value in the fox-specific real-time PCR could be used to determine whether to include a sample in the prevalence estimate. Gradually lowering this cut-off led to higher *E. multilocularis* prevalence estimates based on environmental fecal sampling (Fig. 5A). The number of excluded *E. multilocularis*-positive samples started to decrease strongly if cut-offs <30 in fox DNA PCR were applied to select for samples suitable for prevalence estimation (Fig. 5B). The highest prevalence estimate was reached if a cut-off of Ct <27 was applied (26.3%; 95% CI 17.7–37.2%; Fig. 5A). If the cut-off decreased further, the number of positive and negative samples decreased in parallel (Fig. 5B), while the 95% CI widened (Fig. 5A). This suggests that successful PCR detection of *E. multilocularis* DNA depends on the detectability of minimum concentration of fox-specific DNA, which serves as a proxy for fecal sample quality.

## 4 Discussion

The present study compared prevalence estimates of *E. multilocularis* positive findings as determined by necropsy analysis of hunted animals and examination of fox and raccoon dog fecal samples collected from the environment. Although fecal analysis of environmental samples yielded a lower proportion of positive findings compared to necropsy, it covered a much larger area within the municipalities (Table 5). Host DNA concentration-based sample selection for environmental feces, focusing on samples with higher levels of host-DNA resulted in a proportion of positive *E. multilocularis* findings that more closely matched the proportion of positive findings obtained from fox necropsy.

**Table 5:** Summary on advantages and disadvantages of environmental fecal sampling relative to necropsy analysis for *E. multilocularis* in foxes and raccoon dogs.

Comparison	Environmental feces	Necropsy
Advantages of environmental fecal sampling	∼46.4 km^2^ covered, better representation of communities	∼15.3 km^2^ covered, biased by not-contributing hunters
	Logistically easy	Logistically difficult (−20 °C freezer for storage of carcasses near to hunting area; regular transport; necropsy room)
	Less time consuming, 2.5 months duration (2 person teams, not skilled [∼80 h, 13 tours])	Much more time consuming, 16 months duration (hunting, necropsy [4 person-necropsy team, ∼ 24 h], Sedimentation Counting Technique [1 person, several weeks])
Disadvantage environmental fecal sampling	Detection apparently less sensitive; there is a need to restrict to high quality samples, e.g. based on host-specific real-time PCR results	Detection apparently more sensitive
	Species needs to be determined by PCR	Species does not need to be confirmed
	An individual animal may contribute more than one sample – microsatellite typing necessary, but limited sensitivity of typing	Findings are related to a single animal
	Most likely patent infections are preferentially discovered	Prepatent and postpatent infections can be detected

Necropsy analysis revealed that 37.9% of foxes and 15.4% of raccoon dogs were positive for *E. multilocularis*. However, PCR analysis of environmental fecal samples—excluding a PCR-inhibited sample—yielded estimates of 18.1% for foxes and 10.0% for raccoon dogs. Although the number of animals examined for necropsy was small and the uncertainty high, the difference in foxes was statistically significant. Other researchers have also observed similar discrepancies when comparing prevalence estimates from necropsy and environmental fecal analyses (Knapp et al., 2014; Poulle et al., 2017). The limited apparent sensitivity represents one of the disadvantages of this way of analysis, which overall is logistically easier and much less time consuming than a prevalence estimation based on necropsy (Table 5). Nevertheless, methodologically the analysis of environmental samples is more complex, as it is necessary to determine the animal species, e.g. by multiplex real-time PCR assays, as applied in this study (Table 5).

There are several possible reasons for the observed differences between necropsy and environmental fecal sampling; some of these are discussed in the following.

(i) Firstly, the areas covered by fox and raccoon dog hunting differ from those covered by fecal analysis. This is because one of the hunters active in the study area contributed almost all of the hunted carnivores, while hunters from the other hunting grounds in the study area contributed few or no carnivores. Therefore, only a portion of the total study area could be covered by necropsy analysis (Fig. 1; Table 5). Interestingly in the areas that were covered by hunters, the examination of environmental feces revealed only few *E. multilocularis* positives (Fig. 1) which could have been caused by differences in the periods for hunting and sampling carcasses and the sampling of environmental feces. (ii) While foxes and raccoon dogs were hunted from November 2023 to March 2025, the environmental fecal sampling only covered the period from February 12th, 2025, to April 25th, 2025. The possibility that the prevalence of *E. multilocularis*-infected predators in our study area was lower in late winter or early spring 2025 (i.e. during the environmental fecal sampling period) than, for example, in autumn, could present a potential explanation. However, observations in Switzerland contradict this hypothesis and thus make this explanation unlikely; the highest prevalences of *E. multilocularis* in Swiss foxes were observed in springtime, from March to April (Brossard et al., 2007). (iii) Environmental conditions could have reduced the availability of specific DNA in fecal samples as it has been argued previously (Knapp et al., 2014). In addition, fecal samples may contain inhibitory components which may reduce the sensitivity of PCR (Knapp et al., 2014). However, in our experience the analysis of fecal samples collected during necropsy provide no evidence for an increased susceptibility of our PCR to inhibition (Kunisch et al., 2025). Furthermore, in the duplex real-time PCR used in this study to detect *E. multilocularis* in environmental samples, an internal inhibition control was applied. In only a few samples (*n* = 4) inhibition was observed. Therefore, it appears that environmental influences, including degradation by UV light or leaching by rain, may have been the most likely reasons for less frequent detection of *E. multilocularis* DNA in environmental fecal samples as compared to necropsy analysis (Table 5). We carefully recorded the appearance of samples in detail and found that particularly dark or dominantly dark samples were associated with lower Ct values in the fox-specific PCR, indicating higher levels of DNA available for amplification. This suggests that darker-colored samples were likely exposed to environmental influences for a shorter period, compared to lighter or white-colored samples. The white color likely emerges from the calcium content of the diet, particularly from bones, and becomes more prominent as darker organic matter degrades over time.

When analyzing environmental fecal samples, there is the possibility that individual animals may be sampled multiple times; this may result in an apparent prevalence that is either reduced – in case of negative animals – or increased – in case of positive animals (Table 5). Genotyping of fox DNA in feces has been applied to assign samples to individual animals (Da Silva et al., 2024). Unfortunately, we needed a relatively high fox DNA concentration for genotyping. In a preliminary experiment (data not shown), we observed that only samples with a Ct value <27 in the fox real-time PCR allowed us to genotype foxes in a sufficient number of marker regions. Therefore, only in 41/205 fox samples a number of ≥5 markers could be genotyped and in 35 the sex of the individual could be determined. A potential reason for the low number of genotyped fox fecal samples might be that our DNA isolation protocol was optimized for the detection of *E. multilocularis* DNA. However, for host genotyping, alternative DNA isolation methods have been proposed that enable the recovery of higher concentrations of host DNA for genotyping purposes (Beja-Pereira et al., 2009; Chiou and Bergey, 2018).

None of the genotyped fox fecal samples showed a 100% match with any other sample, which may suggest that all 41 samples came from different foxes. However, when a more lenient arbitrarily selected threshold of at least 75% marker matching was applied and only samples with a sufficient number of amplified markers were considered, we found some evidence of potentially repeated sampling. Such samples could be grouped into clusters based on imperfect microsatellite marker co-occurrences. Assuming that each cluster represented a single animal, further confirmed by the observation that most of the sample pairs had been collected in close proximity to each other in the field, typing results suggested that the 41 typed samples actually may have originated from only 33 foxes. This in turn could suggest that repeated sampling of about one in five samples (i.e. 19.5%) may have occurred in this study. This is much lower compared to a study from France, which was about one in three samples (Da Silva et al., 2024). However, this difference is plausible, as the fecal sampling area in our study was more than 15-times larger and the time period of our study much shorter. Nevertheless, the occurrence of repeated sampling in our study was likely, especially when considering that there was a short distance between sampling sites and that a single fox is estimated to defecate about 8-times a day (Webbon et al., 2004). It remains unclear whether repeated sampling could have influenced the observed frequency of positive fecal findings. In case of a large number of samples originating from a large number of individual animals it is likely that both infected and uninfected foxes were equally affected by repeated sampling and the impact on the observed prevalence is expected to be low. However, if prevalence estimation is based on only few individuals and these are re-sampled several times, prevalence estimates might become strongly biased.

In one pair of samples suspected to originate from the same fox, only one of the fecal samples tested positive for *E. multilocularis*, while the other tested negative. This seems plausible, even if the sample originated from a single animal. Several reasons could have caused a negative result in one and not the other of the two fecal samples that likely originated from the same fox. (i) Fecal samples could have been dropped at different times during the course of the infection (prepatent, early patency, latent patency (Thompson, 2017)). From experimental infections it is known that the likelihood for detecting *E. multilocularis* DNA in fecal samples is highest in patency but also possible in prepatency, which suggests that not only eggs are source of this DNA but also worms or cellular material released by worms (Al-Sabi’ et al., 2007). (ii) In addition, intermittent shedding of eggs but also worms or worm materials could explain our observation; this is well known for *E. multilocularis* eggs, particularly in a later phase of the patency period (Kapel et al., 2006; Nonaka et al., 1996). (iii) *E. multilocularis* or its DNA could have been heterogeneously distributed in the fecal samples and thus evaded detection. (iv) Limitations in analytical sensitivity or inhibition of PCR detection in one, but not the other sample could have been another possible reason for our observation.

Assuming a fox-density of about 1–2 foxes per km^2^ during late winter or early spring (Janko et al., 2012; Webbon et al., 2004) the total population of foxes in the study area covered by environmental fecal sampling was estimated at about 46–92 animals. Under this prerequisite, the number of *n* = 33 successfully genotyped foxes allowed us to conclude that a reasonable number of the foxes present at that time in this remote part of the island Rügen had been sampled, but also that quite a number of the remaining samples in which fox-genotyping was not possible originated from the same population of foxes. Based on the reduced list of genotyped fox samples, 27.3% had tested *E. multilocularis* positive, a proportion which is closer to the proportion of positive foxes observed by necropsy (37.9%), which in turn shows that quality and potentially the ‘freshness’ of environmental fecal samples is an important criterion, for gaining accurate *E. multilocularis* prevalence estimates in fox populations.

Currently, there are no formal criteria for determining whether an environmental fecal sample is suitable for estimating *E. multilocularis* prevalence. To address this, we recorded additional features for each sample and examined their relationship with *E. multilocularis* detection. Despite our efforts to document the appearance of fecal samples as thoroughly as possible, we found it difficult to identify objective criteria for selecting samples suitable for an *E. multilocularis*-PCR analysis. We observed a statistically significantly association between the color of a sample and the likelihood of obtaining a Ct-value <27 in the fox-specific real-time PCR. However, the color of the sample did not correlate with the detection of *E. multilocularis* DNA in the sample.

Interestingly, we observed that gradually decreasing a Ct value-based cut-off in the fox-specific real-time PCR to determine whether a sample should be included for prevalence estimation led to *E. multilocularis* prevalence estimates that were closer to those obtained from necropsy analysis. Excluding samples with low levels of definitive host DNA likely contributes to a more accurate estimate of the *E. multilocularis* prevalence, as it reduces the influence of less reliable or degraded fecal samples in the overall estimate.

Surprisingly we observed that *E. multilocularis* positivity in real-time PCR was statistically significantly associated with unformed or partially unformed environmental fecal samples, while well-formed fox feces were significantly less often positive. It seemed that these feces were already unformed or partially unformed at deposition, and no association with disintegration was seen. In publications on experimental infection of foxes and other definitive hosts, like dogs, no such observations were reported to the best of our knowledge. It is possible that these were overlooked because foxes in these experiments had altered feces differing in form and consistency due to being fed a non-natural diet in captive husbandry conditions, e.g. most likely dog food instead of their natural prey. Recently, *E. multilocularis* infection was reported in several dogs and this observation was associated with diarrhea in some of the animals (Evason et al., 2023; Evason et al., 2025); however, these reports are only anecdotal and additional field studies are needed to confirm such observations.

In the present study, a total of 365 fecal samples were collected. Species diagnostics using real-time PCR revealed that 56.2% of these samples were from foxes. The success rate of identifying fox feces was slightly lower than the rate reported for studies in France (Da Silva et al., 2020; Poulle et al., 2017). In contrast to foxes, feces from raccoon dogs were rarely found, most likely due to the differing defecation behavior of these animals in hidden latrines (Watanabe et al., 2021), which were hard to detect by the fecal sampling teams.

## 5 Conclusion

Our study area focused on two municipalities on the island of Rügen in northeastern Germany, a region that had previously been classified as an area of  low to moderate *E. multilocularis* endemicity. The relatively high prevalence observed in these municipalities suggested definitively endemicity; however, the results of such local studies should not be used to draw conclusions regarding the situation across the entire island or within the federal state of Mecklenburg-Western Pomerania. Nevertheless, our findings underscore the need for enhanced surveillance—including also regions where the endemicity of *E. multilocularis* has not yet been assessed, or was last assessed a long time ago.

Both environmental fecal sampling and necropsy of hunted animals, have advantages but also disadvantages (Table 5). While it usually takes years to determine a prevalence estimate via necropsy analysis, very much due to the time needed to collect the required number of carcasses, environmental fecal sampling in this study immediately provided information on the existence of *E. multilocularis* infection in foxes and raccoon dogs. Difficulties, like limited sample quality can probably be overcome by establishing quality criteria. For instance, in the present study, adjusting the Ct value cut-off in the fox-specific real-time PCR seemed to be reasonable to avoid the prevalence estimate being biased by false negative samples due to leaching and degradation of *E. multilocularis* materials or DNA by environmental influences. In addition, our study showed again, that *E. multilocularis* positive feces were not rare in the environment, even in an area with moderate endemicity, and that these feces represent a risk for humans, which is in accordance with risk factors previously identified for humans. These risk factors include dog or cat ownership (due to the possible role of these animal species as definitive hosts of *E. multilocularis*), living in a rural area, having a kitchen garden and being involved in farming or hunting activities (Conraths et al., 2017; Panel on Animal Health and Welfare, 2015). In addition to humans, companion animals, such as dogs may acquire infection via environmental feces and act as intermediate hosts for alveolar echinococcosis (Corsini et al., 2015; Kolapo et al., 2023).

## CRediT authorship contribution statement

**Josefine Wassermann:** Writing – review & editing, Methodology, Investigation, Formal analysis. **Marin Bussi:** Writing – review & editing, Methodology, Investigation, Formal analysis. **Martina Abs:** Writing – review & editing, Methodology, Investigation, Data curation. **Alrik-Markis Kunisch:** Writing – review & editing, Methodology, Investigation. **Martin J. Oettler:** Writing – review & editing, Methodology, Investigation. **Marie Krebs:** Writing – review & editing, Investigation. **Franziska Rachel:** Writing – review & editing, Methodology, Investigation. **Jonas Heck:** Writing – review & editing, Investigation. **Christine Luttermann:** Writing – review & editing, Methodology, Investigation. **Heiko Schmüser:** Writing – review & editing, Methodology, Investigation. **Kerstin Wernike:** Writing – review & editing, Project administration, Methodology, Investigation, Funding acquisition. **Carola Sauter-Louis:** Writing – review & editing, Project administration, Investigation. **Hannes Bergmann:** Writing – review & editing, Visualization, Methodology, Investigation. **Gereon R.M. Schares:** Writing – review & editing, Writing – original draft, Visualization, Validation, Supervision, Project administration, Methodology, Investigation, Funding acquisition, Formal analysis, Data curation, Conceptualization.

## Consent for publication

Not applicable.

## Ethical approval

All samples have been retrieved from animals that were hunted by local hunters according to the appropriate German legislation. No ethical approval was required for this kind of sample collection in the Federal state of Mecklenburg-Western Pomerania, Germany.

## Funding

The study is part of the One Health project Wildlife disease monitoring in Mecklenburg-Western Pomerania through an integrated One Health surveillance-response system (WiMoPOH) funded by the “Initialisierungs- und Vernetzungsfonds für Infektionsforschung” managed by the Helmholtz Institute for One Health. In addition, this work was co-funded by the European Union's Horizon Europe Project 101136346 EUPAHW. Views and opinions expressed are however those of the authors only and do not necessarily reflect those of the European Union or the European Research Executive Agency. Neither the European Union nor the granting authority can be held responsible for them.

## Declaration of competing interest

The authors declare the following financial interests/personal relationships which may be considered as potential competing interests:

Gereon R. M. Schares reports financial support was provided by Helmholtz Association of German Research Centres. Gereon R. M. Schares reports financial support was provided by European Union. Kerstin Wernike reports financial support was provided by Helmholtz Association of German Research Centres. Kerstin Wernike reports financial support was provided by European Union. If there are other authors, they declare that they have no known competing financial interests or personal relationships that could have appeared to influence the work reported in this paper.

## Data Availability

The data generated and analyzed during this study are available as Supplementary Information or from the corresponding author upon reasonable request.

## Appendix A Supplementary data

Supplementary material 1 Additional file 1: Validation of triplex real-time PCR-I and triplex real-time PCR-II for the identification of the animal species from which environmental feces originated

Supplementary material 2 Additional file 2: Table S1. Results on *Echinococcus multilocularis* for foxes and raccoon dogs, with geographic coordinates of the sampling sites. Additional file 2: Table S2. Comparison of the results obtained for *n* = 84 necropsied foxes and raccoon dogs by Sedimentation Counting Technique (SCT) and duplex real-time PCRs on small intestinal swabs (swab) and feces collected from the terminal rectum (feces). Unweighted Kappa values and their 95% confidence intervals (CI) are provided. Additional file 2: Table S3. Environmental fecal samples: Geographic coordinates of the sampling location, detailed sample characteristics, real-time PCR results for species identification and analysis on *Echinococcus multilocularis,* as well as fox genotyping results.
