# Supplementary material for: How can environmental fecal sampling support Echinococcus multilocularis surveillance in wild carnivores?
Source: Food Waterborne Parasitol. 2026 Jun 27;44:e00352. doi: 10.1016/j.fawpar.2026.e00352 (PMC13351889; doi:10.1016/j.fawpar.2026.e00352)
Supplement: Supplementary material 1 — Additional file 1: Validation of triplex real-time PCR-I and triplex real-time PCR-II for the identification of the animal species from which environmental feces originated [file mmc1.docx]

**Validation of triplex real-time PCR-I and triplex real-time PCR-II** **for the identification of the animal species from which environmental feces originated**

The triplex real-time PCR-I and triplex real-time PCR-II were validated using DNA isolated from feces of red foxes (n = 4), raccoon dogs (n = 4), raccoons (n = 4), wolves (n = 4), domestic dogs (n = 3), wildcats (n = 4), domestic cats (4), badger (n = 3), and pine marten (n = 2).

Primers and probes used in triplex-real-time PCR-I and triplex-real-time PCR-II were published (Knapp et al., 2016 [fox, dog, cat], Koyama et al., 2026 [raccoon dog]) while primers and probes for raccoon dogs had been established by our working group.

The validation experiments revealed that DNA of wolves and wildcats reacted in the real-time PCR previously established for the detection of dogs and cats, respectively (Knapp et al., 2016). The real-time PCR targeting fox showed the highest number of cross-reactions with fecal DNAs from other species (n=7). Because also the other species-specific PCRs showed occasionally some cross-reactivity, Ct-values > 38 were not used to decide on the animal species from which a fecal sample originated. In case, two or more host species-specific real-time PCRs showed reactions, diagnosis was based on which real-time PCR had revealed a Ct value, at least 3 Cts lower than the remaining species-specific real-time PCRs (ΔCt ≥ 3). In the DNAs used for validation the lowest ΔCt was at 6.63 (**Table**).

**References**

Knapp, J., Umhang, G., Poulle, M.L., Millon, L., 2016. Development of a Real-Time PCR for a Sensitive One-Step Coprodiagnosis Allowing both the Identification of Carnivore Feces and the Detection of Toxocara spp. and Echinococcus multilocularis. Appl. Environ. Microbiol. 82, 2950-2958, <https://doi.org/10.1128/AEM.03467-15>.

Koyama, H., Kozakai, C., Matsumura, H., Shibaike, H., 2026. Development of Species-Specific Primers for Detecting Raccoon (Procyon lotor) eDNA from Field Water. Japan Agricultural Research Quarterly: JARQ advpub, <https://doi.org/10.6090/jarq.24J25>.

**Table:** A total number 32 fecal DNAs were used to validate two multiplex real-time PCRS for the species-specific identification of samples from red fox, raccoon dog, dog, cat and raccoon- A ΔCt value of at least 3 was used to decide on the correct species, if in more than a single PCR an amplification was recorded.

|  |  | **Triplex PCR-I** | | **Triplex PCR-II** | | |  |
| --- | --- | --- | --- | --- | --- | --- | --- |
| **Sample-ID** | **Species** | **Target fox** | **Target raccoon dog** | **Target dog** | **Target cat** | **Target raccoon** | **ΔCt value(s)** |
| DNA22/1036 | Red fox | 17.59 | N/A | N/A | N/A | N/A | NA |
| DNA22/1037 | Red fox | 17.38 | N/A | N/A | N/A | N/A | NA |
| DNA22/1038 | Red fox | 26.35 | N/A | N/A | N/A | N/A | NA |
| DNA22/1039 | Red fox | 23.86 | N/A | 42.59 | N/A | 30.49 | 18.73, 6.63 |
| DNA20/144 | Raccoon dog | 36.78 | 24.65 | N/A | N/A | 37.00 | 12.13, 12.35 |
| DNA20/158 | Raccoon dog | 42.27 | 27.82 | N/A | N/A | N/A | 14.45 |
| DNA26/1740 | Raccoon dog | 29.42 | 15.23 | N/A | N/A | N/A | 14.19 |
| DNA26/1741 | Raccoon dog | 31.21 | 16.08 | N/A | N/A | N/A | 15.13 |
| DNA20/146 | Raccoon | 39.1 | 37.17 | N/A | 38.83 | 20.44 | 18.66, 16.73, 18.35 |
| DNA20/148 | Raccoon | N/A | N/A | N/A | N/A | 23.14 | NA |
| DNA20/151 | Raccoon | N/A | 37.25 | N/A | N/A | 17.58 | 19.67 |
| DNA20/152 | Raccoon | N/A | N/A | N/A | N/A | 18.07 | NA |
| DNA20/167 | Wolf | N/A | N/A | 33.79 | N/A | N/A | NA |
| DNA20/168 | Wolf | N/A | N/A | 23.56 | N/A | N/A | NA |
| DNA20/169 | Wolf | N/A | N/A | 28.05 | N/A | N/A | NA |
| DNA20/170 | Wolf | N/A | N/A | 25.21 | N/A | N/A | NA |
| PE20/165 | Domestic dog | N/A | N/A | 31.62 | N/A | N/A | NA |
| DNA26/47 | Domestic dog | N/A | N/A | 33.23 | N/A | N/A | NA |
| DNA26/48 | Domestic dog | N/A | N/A | 34.38 | N/A | N/A | NA |
| DNA22/2995 | Wildcat | N/A | N/A | N/A | 17.29 | N/A | NA |
| DNA22/2996 | Wildcat | N/A | N/A | N/A | 15.05 | N/A | NA |
| DNA22/2997 | Wildcat | N/A | N/A | N/A | 16.96 | N/A | NA |
| DNA22/2998 | Wildcat | N/A | N/A | N/A | 17.83 | N/A | NA |
| PE22/166 | Domestic cat | N/A | N/A | N/A | 24.07 | N/A | NA |
| PE20/167 | Domestic cat | N/A | N/A | N/A | 32.44 | N/A | NA |
| DNA26/44 | Domestic cat | N/A | N/A | N/A | 27.79 | N/A | NA |
| DNA26/45 | Domestic cat | N/A | N/A | N/A | 27.17 | N/A | NA |
| 2025BVD03744 | Badger | N/A | N/A | N/A | N/A | N/A | NA |
| 2025BVD03745 | Badger | N/A | N/A | N/A | N/A | N/A | NA |
| 2025BVD03757 | Badger | 39.59 | N/A | N/A | N/A | 39.32 | NA |
| DNA20/153 | Pine marten | N/A | N/A | N/A | N/A | N/A | NA |
| 2024BVD12663 | Pine marten | 41.11 | N/A | N/A | N/A | N/A | NA |
